# Acoustic deterrents influence foraging activity, flight and echolocation behaviour of free-flying bats

**DOI:** 10.1242/jeb.242715

**Published:** 2021-10-28

**Authors:** Lia R. V. Gilmour, Marc W. Holderied, Simon P. C. Pickering, Gareth Jones

**Affiliations:** 1 School of Biological Sciences, University of Bristol, Bristol BS8 1TQ, UK; 2 Department of Applied Sciences, Faculty of Health and Applied Sciences, University of the West of England, Bristol BS16 1QY, UK; 3 Ecotricity Group Limited, Stroud GL5 3AX, UK

**Keywords:** Thermal imaging, Videogrammetry, Ultrasound, Chiroptera, Flight-path tracking

## Abstract

Acoustic deterrents have shown potential as a viable mitigation measure to reduce human impacts on bats; however, the mechanisms underpinning acoustic deterrence of bats have yet to be explored. Bats avoid ambient ultrasound in their environment and alter their echolocation calls in response to masking noise. Using stereo thermal videogrammetry and acoustic methods, we tested predictions that: (i) bats would avoid acoustic deterrents and forage and social call less in a ‘treated airspace’; (ii) deterrents would cause bats to fly with more direct flight paths akin to commuting behaviour and in line with a reduction in foraging activity, resulting in increased flight speed and decreased flight tortuosity; and (iii) bats would alter their echolocation call structure in response to the masking deterrent sound. As predicted, overall bat activity was reduced by 30% and we recorded a significant reduction in counts of *Pipistrellus pygmaeus* (27%), *Myotis* spp. (probably *M. daubentonii*) (26%), and *Nyctalus* spp. and *Eptesicus* spp. (68%) passes. *Pipistrellus pygmaeus* feeding buzzes were also reduced by the deterrent in relation to general activity (by 38%); however, social calls were not (only 23% reduction). Bats also increased their flight speed and reduced the tortuosity of their flight paths, and *P. pygmaeus* reduced echolocation call bandwidth and start frequency of calls in response to deterrent playback, probably owing to the masking effect of the sound. Deterrence could therefore be used to remove bats from areas where they forage, for example wind turbines and roads, where they may be under threat from direct mortality.

## Abstract (precis)

**Highlighted Article:** Using novel flight path tracking and acoustic methods, we show that bats alter their activity, foraging and echolocation behaviour in response to an acoustic deterrent.

## INTRODUCTION

Acoustic deterrents reduce bat activity at foraging sites in the UK (Gilmour et al., 2020), reduce bat mortality at wind farms (Arnett et al., 2013; Weaver et al., 2020) and are effective in tackling human–bat conservation conflicts in historic buildings (Zeale et al., 2016). However, the mechanism for acoustic bat deterrence has not yet been explored. Understanding how acoustic deterrence works and its impact on bats is therefore important for its safe and appropriate use.

Acoustic deterrence systems can be considered as being analogous to a noise disturbance encountered in an animal's environment, for example from natural and/or anthropogenic sources (Schaub et al., 2008; Bunkley et al., 2015; Luo et al., 2015). Potential mechanisms for the effect of noise on animals include noise avoidance, a reduction in attention owing to the noise, and auditory masking (Chan et al., 2010; Purser and Radford, 2011; Francis and Barber, 2013; Moore, 2013; Luo et al., 2015). Noise avoidance usually occurs when a sound in an animal's environment represents an uncomfortable or aversive stimulus or potential stressor (Francis and Barber, 2013; Luo et al., 2015). For example, foraging was reduced in Daubenton's bats (*Myotis daubentonii*) by traffic noise playbacks that did not overlap in frequency with returning echolocation echoes and therefore represented an aversive stimulus, rather than a masking one (Luo et al., 2015).

Reduced attention owing to noise occurs when an animal's ability to focus on important tasks such as foraging or predator avoidance are impaired by another sound source (Barber et al., 2003; Chan et al., 2010; Purser and Radford, 2011; Luo et al., 2015). For example, three-spined sticklebacks (*Gasterosteus aculeatus*) made more food handling errors, resulting in a reduction in foraging efficiency in response to noise, and Caribbean hermit crabs (*Coenobita clypeatus*) were more vulnerable to predation in response to boat noise (Chan et al., 2010; Purser and Radford, 2011). Auditory masking is where the perception of a sound is affected by another masking sound and the threshold level for hearing the original sound is increased by the presence of the second sound (Moore, 2013). For example, wild superb fairy-wrens (*Malurus cyaneus*) were less likely to flee alarm calls in the presence of overlapping high amplitude noise, but not in response to non-overlapping noise (Zhou et al., 2019).

Ambient sound can therefore have a range of impacts on an animal's ability to carry out important behaviours, such as communicating with conspecifics, social behaviour, courtship, foraging and avoiding predators (Schaub et al., 2008; Chan et al., 2010; Bunkley et al., 2015; Mahjoub et al., 2015; de Jong et al., 2018; Tidau and Briffa, 2019; Zhou et al., 2019). However, all three mechanisms are not mutually exclusive, and teasing apart underlying reasons for noise effects can be difficult (Luo et al., 2015).

Previous research on deterrence has alluded to a specific masking effect of the acoustic deterrent stimulus on the echolocation system of bats, precluding their ability to hunt and find prey (Arnett et al., 2013). Along with the passive hearing system of most other vertebrates, bats possess an active hearing system and rely on echolocation to orientate and hunt their insect prey at night (Griffin et al., 1960). Bats are therefore susceptible to another level of auditory masking, often called ‘jamming’, in which sounds from echolocating conspecifics or other ambient sources interfere with returning echoes from their own signals (Griffin et al., 1963). Indeed, bats will often alter their spectral and/or temporal echolocation characteristics in response to jamming by conspecifics, in what is often called a jamming avoidance response (Gillam et al., 2006; Amichai et al., 2015). Bats will also alter their echolocation calls in response to ambient noise, from natural sources such as insect sounds and anthropogenic sources such as traffic noise or gas compressor stations (Gillam and McCracken, 2007; Bunkley et al., 2015). Not all responses to noise in bats are due to jamming of echolocation calls. Masking can also occur when prey-generated sounds necessary to locate prey by gleaning bats are masked by noise (Schaub et al., 2008). Bats may also avoid noise if it represents an uncomfortable or stressful stimulus (Luo et al., 2015). Bat responses to sounds that are overlapping and non-overlapping with their echolocation call frequency range can therefore be used to determine whether the mechanism for noise avoidance is auditory masking, simple aversion, or indeed both (Luo et al., 2015). Masking usually results in a shift in echolocation signal structure in response to a sound, whereas no effect on call structure would be evident if bats are simply avoiding a stressful stimulus (Luo et al., 2015). Bat communication can also be affected by masking, although this has been studied less (Song et al., 2019).

Flying vertebrates such as bats are constrained by the energetic costs of flight and therefore adjust their flight pattern in order to minimise energy expenditure where possible (Pennycuick, 1975; Rayner, 1999; Grodzinski et al., 2009). Foraging bats thus tend to fly at reduced speeds and with more tortuous flight paths (taking a longer and more twisted or convoluted route to reach the same point), compared with when commuting, where their flight paths are faster and more direct (Jones and Rayner, 1989; Grodzinski et al., 2009; Holderied and Jones, 2009). Commuting bats aim to reach their foraging territories quickly, avoiding predation, and therefore fly at higher speeds, using more energy. Once foraging and searching for insects, which represent a patchy resource, bats will forage with slower and more tortuous flight, which is also more energy efficient. Flight speeds and other flight path characteristics can therefore be used to investigate the effect of specific environmental conditions, such as light or noise, on bat behaviour (Polak et al., 2011). For example, flight speeds of *Pipistrellus kuhlii* and *Eptesicus bottae* were significantly increased in floodlit areas compared with natural darkness (Polak et al., 2011). *Eptesicus bottae* also did not forage in the light and flew closer to commuting speed when passing through the beam.

Animal flight speed, tortuosity and other trajectory characteristics are often measured using three-dimensional flight path tracking methods (Dell et al., 2014; Betke et al., 2017). Flight paths of bats have been reconstructed using stereo photogrammetery, stereo videogrammetry, GPS tracking systems and acoustic tracking systems, for example (Jones and Rayner, 1988; Jones, 1995; Holderied et al., 2005, 2008; Hristov et al., 2008; Grodzinski et al., 2009; Polak et al., 2011; Yang et al., 2013; Giuggioli et al., 2015). As bats generally fly at night, visual methods of flight path tracking have often been limited to using a relatively small lit-up area, often using multiflash photography (Jones and Rayner, 1988; Jones, 1995; Polak et al., 2011; Giuggioli et al., 2015). Thermal imaging methods allow the visualisation of animals in dark environments potentially over larger scales (Hristov et al., 2008) and avoiding potential disturbances that may be caused by flashgun lights. Some studies have used thermal imaging for two-dimensional flight path tracking, for example in studying emergence patterns of bats from caves and at offshore wind turbine sites (Hristov et al., 2008; Matzner et al., 2015). However, only a handful of studies have utilised both stereo videogrammetry and thermal imaging methods to study bats to date (Hristov et al., 2008; Yang et al., 2013).

Therefore, we aimed to explore the potential mechanisms underpinning acoustic deterrence and its impact on bats, using a combination of stereo thermal videogrammetry and acoustic recording techniques. In this study, we test the predictions that bats will avoid acoustic deterrents, resulting in a decrease in (i) activity, (ii) foraging and (iii) social behaviour. We also predict bat flight performance will change in response to the deterrent, including (iv) an increase in mean trajectory flight speed and (v) a decrease in tortuosity, owing to a decrease in foraging and more in line with commuting flight. We also predict that (vi) bats at experimental sites will alter their echolocation call structure to avoid auditory masking by the deterrent sound, which overlaps in frequency with bat signals.

## MATERIALS AND METHODS

### Site selection and experimental procedure

We carried out experiments in July and August 2018 at three riparian locations along the River Teme, Knighton, Powys (>5 km apart, to minimise the chances of recording the same bats), selected because of high levels of bat activity. Each site had an area of still water next to a bridge and was flanked by hedges along one side, either along the river or perpendicular to the bridge. We filmed bats flying over the river using two synchronised thermal imaging cameras (Optris PI640 thermal imaging cameras, 640×480 pixel resolution, 33 deg lenses; Optris, Berlin, Germany), Optris PI Connect software (Optris) and a laptop computer (Dell XPS 15, Dell, Austin, TX, USA), recording in .avi uncompressed video format at 32 frames s^−1^. We recorded for 1 h per night, for three nights at each site (9 h of footage in total), starting at approximately 0.5 to 1 h after sunset, when bat activity was highest at these sites. We alternated 5 min silent control periods and 5 min of ultrasonic speaker (deterrent), totalling 6×10 min time blocks of control and treatment over the 1-h experiment. We started each experiment night with a control period, in order to monitor bat activity levels and decide when to start filming. To control for time of night effects, we included time block order as a fixed effect in statistical analysis.

We placed the two Deaton ultrasonic speakers (Deaton Engineering, TX, USA) on ladders at ∼2 m high and ∼15 m behind the cameras, so that the sound field covered ∼15–30 m in the *z* plane of the cameras' field of view (FOV). Speakers were the same as used at wind energy facility in North America (Arnett et al., 2013), in churches (Zeale et al., 2016) and foraging sites in the UK (Gilmour et al., 2020). Speakers comprised 16 transducer units (SensComp 600 series, SensComp, MI, USA; see http://rfelektronik.se/manuals/Datasheets/SensComp600.pdf for typical beam pattern) that emitted ultrasound at a frequency range of 20–100 kHz, with a frequency of maximum energy (FmaxE) at 50 kHz that overlapped with the echolocation call FmaxE of bat species likely to be common at the sites in this study, including *Pipistrellus pipistrellus* (45 kHz), *P. pygmaeus* (55 kHz), *Nyctalus* spp. and *Eptesicus* spp. (25–30 kHz), and *Myotis* spp. (30–50 kHz) (see Gilmour et al., 2020 for power spectrum and spectrogram of output). Speaker sound pressure levels were calculated to be 98 dB SPL at 1 m, 52 dB SPL at 15 m and 21 dB SPL at 30 m, at 50 kHz (assuming 14°C, 90% relative humidity and 101.325 kPa) (Fig. S1). Beyond 40 m, the sound pressure level was calculated as below 3 dB SPL for 50 kHz.

Pilot work at the same sites allowed us to predict that there would be approximately a 60% reduction on average in bat activity (flight paths) over the 15–30 m treatment area. We therefore chose the speaker positions to increase the likelihood of recording an effect, but also to ensure that there were bats present that could respond to the deterrent present in the treatment area. We positioned the two cameras at the same height (1 m), 4 m apart, parallel to the ground (using a spirit level), so that the inner edge of their FOV overlapped at 3.2 m and the side edges of their FOV were parallel, in order to maximise the combined FOV and treatment area covered (4–5 m wide, depending on distance) (Fig. S2).

All equipment was powered by a low-noise generator (Honda EU10i, Honda, Tokyo, Japan) that did not emit ultrasound and was unlikely to affect bat activity (Stone et al., 2009). The generator was placed at least 10 m away from the treatment area and ran during both control and treatment periods. We also placed an SM2BAT+ detector and SMX-US microphone (Wildlife Acoustics, MA, USA; continuous .wav recording; 384 kHz sampling rate; SNR 10) next to the bridge at ∼30 m from the deterrent, angled towards the water at each site, to record bat calls in the treatment zone during the experiment hour, but also far enough away to avoid masking effects of the deterrent on recordings.

### Calibration

We carried out calibration of the treatment area (extrinsics) and the camera lens parameters (intrinsics) using a bespoke commissioned calibration target, comprising an aluminium cross with thirty 11 mm diameter tungsten bulbs (1.5 V, 300 mA) wired in parallel on four arms, powered by four D cell batteries (Fig. S3). Three of the arms were identical with eight bulbs, and one arm had six bulbs arranged in three pairs, to allow its identification in the thermal footage. The target was thermally insulated using heating insulation board and a cardboard panel, so the heat signature from the person carrying the target was obscured as much as possible.

We calculated bulb coordinates and filmed the calibration target in eight different orientations and at three angles (24 positions) on each repeat night and extracted image stills using VirtualDub (v. 1.10.4, Free Software Foundation, Cambridge, MA, USA). Images were prepared using Photoshop CS5 Extended (v.12, Adobe Systems, San Jose, CA, USA) threshold, curves and paint brush functions, ready for extraction of *in situ* bulb coordinates from each camera using MatchPoint 1.0 (software developed using original target bulb coordinates and MATLAB (v.R2017a, 9.2.0.556344, MathWorks, Natick, MA, USA) especially for this application). We extracted camera intrinsics and extrinsics of each nightly set-up using code based on the stereo vision toolbox in MATLAB and used these to plot bat trajectory data.

### Reconstructing trajectories

**Fig. 1.**
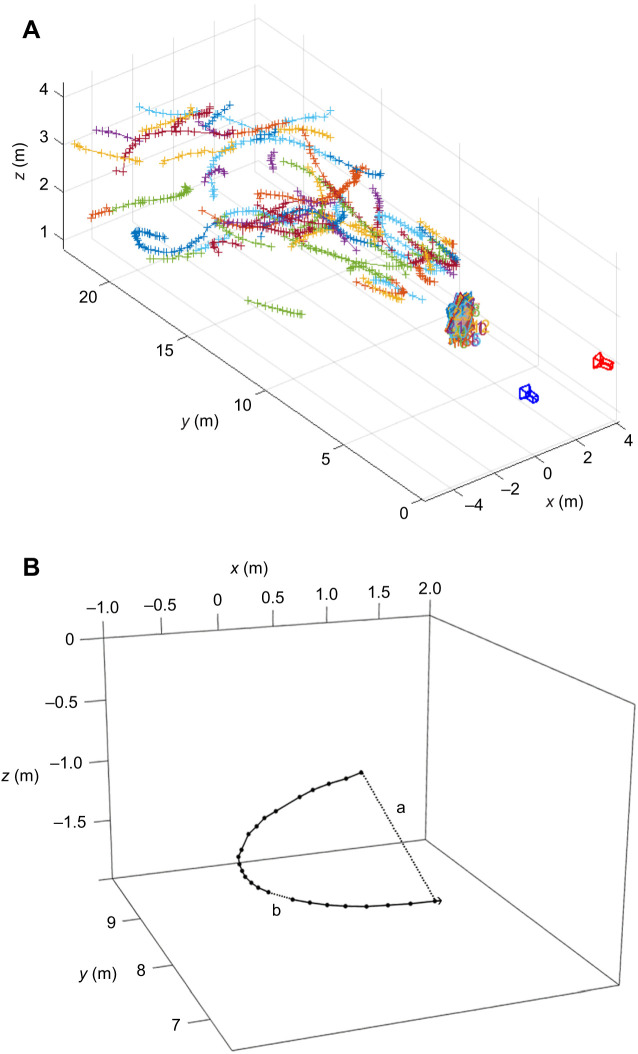
**Example of bat flight trajectories.** (A) Stereo reconstruction created in MATLAB of all possible trajectories in an example 30 s of footage during a silent control period. Cameras are plotted in blue and red. Calibration target positions are also plotted in front of the cameras as 24 separate calibration target coordinates from intrinsic/extrinsic parameter calculations. (B) A single trajectory, showing calculation of trajectory variables from three-dimensional localization points. We show (a) net displacement, calculated as the distance from the first to last localisation (long dotted line), and (b) distance segment (short dotted line between two localisations), used to calculate the total distance, as the sum of all segments. Distance (m) and height (m) from the cameras were also calculated as the mean of *y* and *z* localisation coordinates, respectively.

We extracted individual bat localisation coordinates for the two cameras using background subtraction between individual frames of 32 frames s^−1^ video recordings in MATLAB. All possible localisations were then paired and sorted into potential trajectories using defined parameter values, including a maximum speed of 15 m s^−1^, a maximum frame gap (number of frames in between localisations) of 15, a maximum distance between localisations of 1000 mm and minimum trajectory length (number of localisations making up a trajectory) of six (Fig. 1).

Before trajectory coordinates were extracted for each night, we determined the best pixel threshold that allowed the maximum number of trajectories to be determined with the minimum amount of noise. A pixel threshold that was too low could result in insect trajectories or erroneous localisations being plotted and a threshold that was too high would reduce the ability to detect bats, especially further away from the cameras. We selected trajectories that were likely to be true pairings, including those that did not share more than three localisation points with other trajectories. We discarded trajectories that were likely to be erroneous pairings (by comparing *x* and *y* coordinates from both cameras), those that had inconsistent speeds (a difference of >3 m s^−1^ between subsequent localisations) and/or distorted trajectories that did not appear to follow a smooth pathway and had <10% erroneous localisations. Some replication and distortion of individual localisations in a trajectory was still possible with selected trajectories. We therefore took a conservative approach to minimise the likelihood of false positives, which could introduce noise and possibly bias into the dataset. We removed individual distorted and replicated localisations from trajectories that were likely to be true pairings by comparing the trajectory plot and speed calculations. However, this smoothing method was only applied to trajectories where it was obvious there was an erroneous point amongst enough other true localisations (<10% of all localisations). Removing too many potentially erroneous points from a trajectory could bias the data extracted and therefore it would be better to exclude the whole trajectory than risk including one where more localisations were removed than preserved.

### Calculating trajectory variables

We calculated instantaneous flight speed in m s^−1^ as the distance between two localisations in three dimensions, divided by the time between frames (when each frame was 0.032 s) and mean trajectory flight speed (m s^−1^) as the mean of all instantaneous speeds for a single trajectory. We calculated distance from the deterrents (m) as the mean distance from the beginning of the camera FOV to each localisation, i.e. the mean of the *y* coordinate (Fig. 1), plus 15 m owing to deterrent location behind cameras. We calculated height (m) above or below the cameras using the mean of the *z* coordinate for each localisation. We calculated trajectory length as the total travelled distance [the sum of all the distance segments (m) travelled between localisations] and tortuosity value as the ratio of the total travelled distance and net displacement [the distance (m) from the first to the last localization coordinate], divided by 10 (to make it bound between 0 and 1 for statistical analysis).

### Acoustic data collection

We split bat calls recorded for each experiment hour (for nine nights at three sites) into 10 s files and for each file manually identified bat echolocation, feeding buzz and social call data to species or genus level in BatSound 4 (v4.1.4, Pettersson, Uppsala, Sweden; FFT size: 1024; FFT window: Hanning). Species identified from calls included *Pipistrellus pipistrellus* (Schreber 1774), *Pipistrellus pygmaeus* (Leach 1825), *Myotis* spp., *Nyctalus* spp. and *Eptesicus* spp., *Barbastella barbastellus* (Schreber 1774), *Plecotus auritus* (Linnaeus 1758) and *Pipistrellus nathusii* (Keyserling & Blasius 1839). *Myotis* spp. echolocation calls were identified to genus level, owing to the similar nature of their broadband frequency modulated calls; however, in this habitat, most *Myotis* spp. present were likely to be *Myotis daubentonii* (Kuhl 1817), which generally feeds over water (Jones and Rayner, 1988; Russ, 2012). *Nyctalus* spp. and *Eptesicus* spp. were also grouped owing to their similar long-range echolocation calls and flight behaviour, but were likely to be mainly *Nyctalus noctula* (Schreber 1774) (Jones, 1995; Russ, 2012). We identified a new bat pass as a sequence of echolocation calls >1 s from the last pass and feeding buzzes as a short sequence of calls, characterized by a sudden transition to a high repetition rate (Russ, 2012). Social calls are discernible owing to their characteristic shape and lower frequency and can also be identified to species or genus level in most cases (Pfalzer and Kusch, 2003; Middleton et al., 2014). We took the sum of counts from 10 s files to get an overall pass/feeding buzz/social call count for each 5-min time block. We added the deterrent noise level recorded during noise playback to control files using the ‘sum wave files’ function in MATLAB before analysis, so as not to introduce bias from control files being easier to score.

### Call parameter measurements

We extracted call measurements for 450 calls from 150 passes (three calls per pass) for each control and treatment pair in 10-min blocks (300 passes with 900 calls in total) using BatSound 4 (v4.1.4, Pettersson). We extracted measurements from search phase calls only, as approach phase calls have a more broadband structure, a higher frequency of maximum energy and an increasingly shorter pulse interval, as the bat approaches the prey (Griffin et al., 1960). Single bat passes were rare at these busy riparian habitats, so we included passes where one or two bats were present simultaneously but controlled for number of bats present in the model specification (see Statistical analysis, below).

For each call, we manually measured frequency of maximum energy (kHz) using the power spectrum function and end and start frequency (kHz) using the spectrogram and measurement cursor in BatSound 4. To avoid the effect of attenuation of high frequencies on calls recorded at distance, we used a cut-off in amplitude of a call of >15% (using the oscillogram window in BatSound 4). We also used the same spectrogram settings for every file (threshold 0; amplitude contrast 0; frequency resolution 525 Hz; FFT size 1024 samples; FFT window: Hanning; time between FFTs 2.7 ms). We calculated bandwidth (kHz) as the difference between start and end frequency. We also measured call duration as the time (ms) from the beginning to end of the call and pulse interval as the time (ms) from the beginning of one call in a pass to the start of the next.

### Statistical analysis

#### Trajectory measurement data

We analysed trajectory measurement data using linear mixed-effect models (LMMs) using the R (v3.5.2) package lme4 (v.1.1-19) (https://CRAN.R-project.org/package=lme4) and generalized linear mixed-effect models (GLMMs) using glmmTMB (v.0.2.3) (Brooks et al., 2017), depending on the distribution of the response variable. Flight speed and distance followed a Gaussian distribution and were therefore analysed using an LMM, whereas height and length required a transformation to be able to carry out an LMM (Box–Cox and log transformations, respectively). Tortuosity value data followed a continuous proportional distribution between 0 and 1 and therefore were analysed using a beta binomial GLMM with a cloglog link function.

Full models all contained the fixed effects deterrent treatment (levels: deterrent/control), time block order (5-min blocks), flight distance (m) and an interaction term between deterrent treatment and distance (apart from the distance model, which only contained deterrent treatment and time block number). We retained the random effect structure of time block (*N=*12) nested in night (*N=*9), nested in site (*N=*3) in all models. Final models were selected based on second-order Akaike’s information criterion (AICc), where a difference in AICc of >2 between a model and a nested model indicated a better fit. Estimates, s.e. and *t*/*z* values were obtained from model summaries, and χ^2^, d.f. and *P*-values were calculated from likelihood ratio tests (LRTs) between a model containing a term, and a nested model without that term (or the null model). Model selection statistics (AICc values) for all models are presented in Tables S1–S3. We validated models and checked for heteroskedasticity, overdispersion and zero inflation using simulation functions and residual plots in the Dharma package (https://CRAN.R-project.org/package=DHARMa) in R.

#### Acoustic data

We analysed acoustic bat call data in R using the same method as above, with Poisson or negative binomial GLMMs using the lme4 package (https://CRAN.R-project.org/package=lme4), depending on the distribution of the response variable. Response variables included counts of passes, feeding buzzes and social calls of *P. pygmaeus*, and passes of *Myotis* spp., *P. pipistrellus* and a group containing *Eptesicus serotinus* and *Nyctalus* spp. There were not enough data to analyse passes, feeding buzzes or social calls from any other species (<20 passes per night for majority of nights for a site). We excluded the first minute of each block in analysis, to minimise spillover effects from previous blocks and make sure each block had the same number of files analysed. Therefore, we included twenty-four 10 s files (4 min) from each 5-min time block in the analysis.

#### Call parameter data

We analysed call parameter data using the same methods as the acoustic data analysis, but instead using LMMs in lme4 (Bates et al., 2015), owing to the Gaussian distribution of all response variables. Response variables included bandwidth, start and end frequency (kHz), frequency of maximum energy (kHz), and duration and pulse interval (ms). We included the fixed effects deterrent treatment, time block order and number of bats (number of bats present simultaneously in a pass). We included the random effects of site, night, block pair, time block and call sequence number (arranged in a nested design). Block pair was included owing to the differing numbers of passes analysed for different time block pairs (a block pair consisted of two 5-min time blocks, one control and one treatment). Call sequence number was included to identify the pass number, from each time block, for each call. A Bonferroni correction was applied to *P-*values obtained from LRTs for each parameter, to control for multiple testing on the same call data. Both adjusted and non-adjusted *P-*values are presented in the Results.

#### Ethics statement

This study was carried out under ethical approval by the University of Bristol Animal Welfare and Ethical Review Body (licence no: UB/17/045). It was also carried out under licence with strict recommendations from the government licensing departments Natural England (2015-12272-SCI-SCI) and Natural Resources Wales (66141:OTH:CSAB:2015). Privately owned sites were accessed with permission for all field experiments.

## RESULTS

### Bat flight trajectory measurements

We extracted measurements from 1167 viable flight trajectories from a total of 9 h of footage, recorded on nine nights at three sites (1 h per night, three nights per site), including 284 recorded when the deterrent speakers were on and 883 from silent control periods. Trajectories ranged from 0.38 to 11.23 m in length, with a mean (±s.d.) length of 2.56±1.63 m, and contained 6–65 trajectory segments (14.65±8.00). Bat trajectories were recorded from the beginning of the stereo FOV at 5.47 m from the cameras, up to the limit of the cameras' FOV at 26.82 m, at a mean distance of 14.24±3.98 m from the beginning of the cameras’ FOV, equating to a range of ∼20–40 m from the deterrent speakers. Bat flight height ranged from 2.59 m below the camera line of sight to 5.77 m above, 0.46±1.56 m on average.

**Fig. 2.**
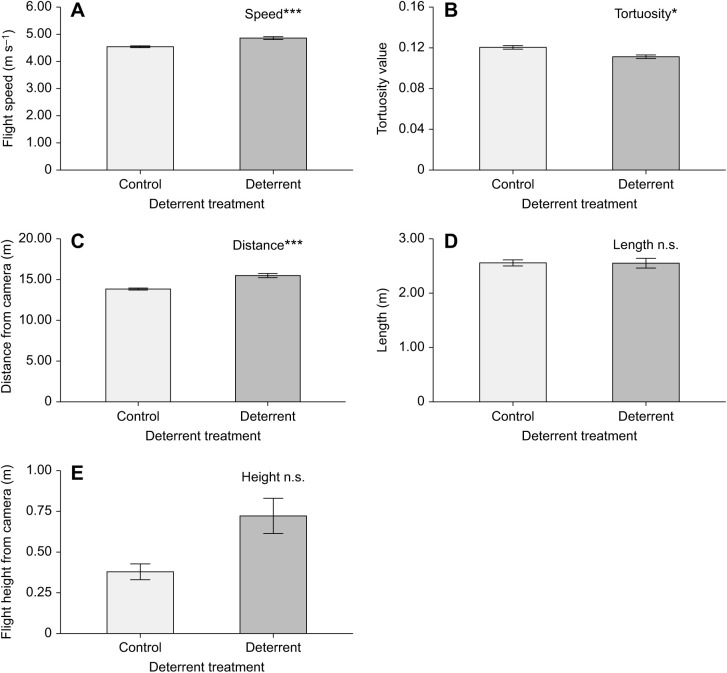
**Boxplots of flight trajectory measurements, during silent control (white) and deterrent (shaded) periods.** (A) Flight speed, (B) tortuosity value and (C) distance from deterrents. Data from a total of nine nights (three nights at three sites, with each site containing six 10 min time blocks of alternating 5 min control and treatment periods). Boxes include 25th percentile, median and 75th percentiles, with 95% confidence interval depicted as whiskers, *P-*values from likelihood ratio tests comparing LMM/GLMMs with and without deterrent treatment (all plots) and/or time block (A) only (*P<0.05, ***P<0.001).

Bat flight speed per trajectory ranged from 1.76 to 7.99 m s^−1^ (mean 4.62±0.98 m s^−1^) and tortuosity value ranged from 0.03 to 0.95 (0.12±0.05). During playback, bat trajectory speeds were significantly higher (4.86±0.92 versus 4.54±0.99 m s^−1^), less tortuous (0.11±0.03 versus 0.12±0.05) and at greater distances (15.49±4.39 versus 13.84±3.76 m) from the deterrents compared with when no sound was played (Table 1, Fig. 2; for model selection statistics, see Table S1). Bat speed significantly decreased over the experiment hour and tortuosity increased with increased distance from the deterrents (Table 1, Fig. 2A). There was no effect of the deterrents on flight height (boxLMM: χ^2^=0.036, d.f.=1, *P*=0.85), or trajectory length (logLMM: χ^2^=0.80, d.f.=1, *P*=0.37).

**Table 1.**
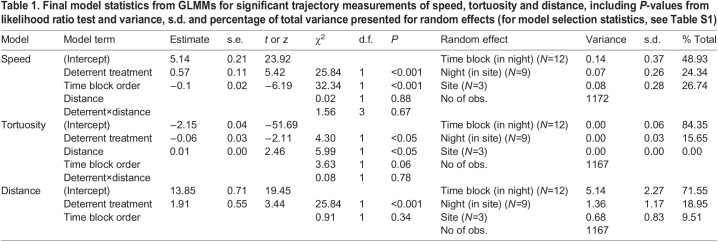
Final model statistics from GLMMs for significant trajectory measurements of speed, tortuosity and distance, including *P*-values from likelihood ratio test and variance, s.d. and percentage of total variance presented for random effects (for model selection statistics, see Table S1)

### Echolocation call analyses

We identified 5440 bat passes, 1343 feeding buzzes and 718 social calls from a total of 9 h of ultrasonic recording, on nine nights at three sites (1 h per night, three nights per site). Per night, we found means (±s.d.) of 604.44±141.00 passes, 149.22±97.24 feeding buzzes and 79.78±56.94 social calls. Most passes were identified as *P. pygmaeus* (4304, 79.12%), followed by *Myotis* spp. (727, 13.36%) and *P. pipistrellus* (323, 5.94%)*.* The remaining 1.58% of passes comprised *E. serotinus* and *Nyctalus* spp., *B. barbastellus*, *P. auritus* and *P. nathusii*. Species composition was similar during control and deterrent treatment periods (Table 2). Most feeding buzzes and social calls were from *P. pygmaeus* (1243, 92.55% and 703, 97.91% respectively), with the remaining feeding buzzes coming from mainly *Myotis* spp. (78, 5.81%) and social calls from *Myotis* spp., *P. pipistrellus* and the *E. serotinus* and *Nyctalus* spp. group (2.09%).

**Table 2.**
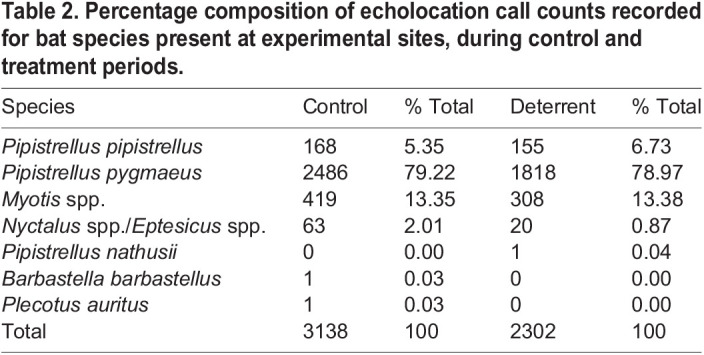
Percentage composition of echolocation call counts recorded for bat species present at experimental sites, during control and treatment periods.

**Fig. 3.**
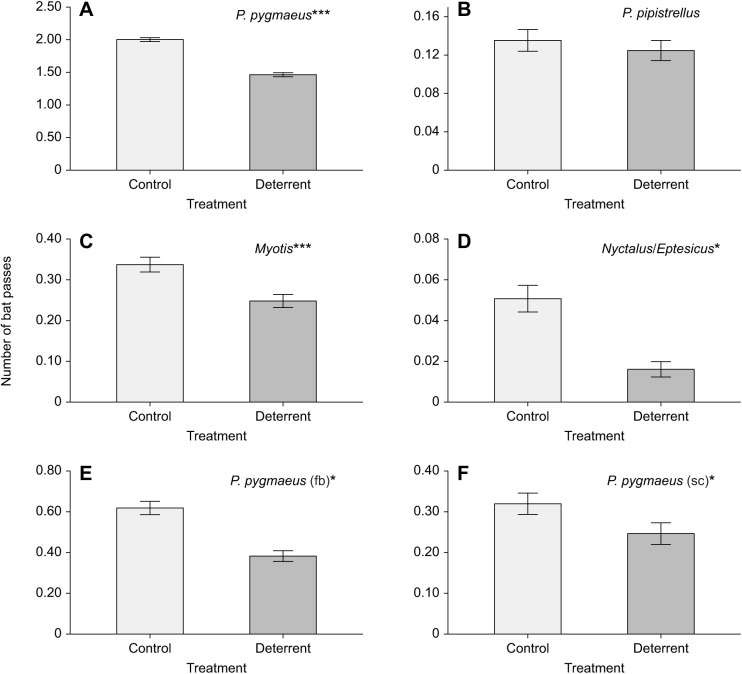
**Mean±s.e.m number of bat passes recorded during silent control (light grey bars) and deterrent (dark grey bars) treatments.** Including data from a total of nine nights (three nights at three sites, with each site containing six 10 min time blocks of alternating 5 min control and treatment periods). Species groups include: (A) *Pipistrellus pygmaeus*, (B) *P. pipistrellus*, (C) *Myotis* spp., (D) *Nyctalus* spp./*Eptesicus* spp., (E) number of feeding buzzes (fb) and (F) social calls (sc) for *P. pygmaeus.* Significant effect of deterrent treatment from GLMM analysis is indicated by asterisks (*P<0.05, ***P<0.001).

Acoustic deterrents reduced the combined number of bat passes, feeding buzzes and social calls by 28.39%. Controlling for time of night, the number of passes of *P. pygmaeus*, *Myotis* spp., and *Nyctalus* spp. and *Eptesicus* spp. were significantly reduced by deterrent treatment (Table 3; see Table S2 for model selection statistics). There were 26.87% and 26.49% fewer *P. pygmaeus* and *Myotis* spp. passes and 68.25% fewer *Nyctalus* spp. and *Eptesicus* spp. passes recorded during deterrent than control periods (Fig. 3A,C,D). However, there was no effect of the deterrent on *P. pipistrellus*. The deterrents significantly reduced the number of *P. pygmaeus* feeding buzzes and social calls (38.15% and 22.92% reduction, respectively) (Table 4, Fig. 3E,F). There were not enough data to model for any other species passes, feeding buzzes or social calls (<20 passes per night for majority of nights).

**Table 3.**
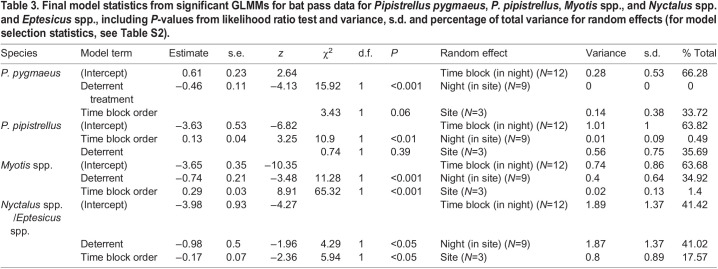
Final model statistics from significant GLMMs for bat pass data for *Pipistrellus pygmaeus*, *P. pipistrellus*, *Myotis* spp., and *Nyctalus* spp. and *Eptesicus* spp., including *P*-values from likelihood ratio test and variance, s.d. and percentage of total variance for random effects (for model selection statistics, see Table S2).

**Table 4.**
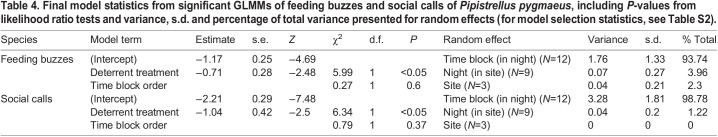
Final model statistics from significant GLMMs of feeding buzzes and social calls of *Pipistrellus pygmaeus*, including *P*-values from likelihood ratio tests and variance, s.d. and percentage of total variance presented for random effects (for model selection statistics, see Table S2).

*Pipistrellus pygmaeus* passes, feeding buzzes and social calls were similar in number throughout the experiment hour and time block was not significant (Tables 3 and 4). There were more *Myotis* spp. and *P. pipistrellus* passes in the latter half an hour and time block was significant (Table 3). There were also significantly more *Nyctalus* spp. and *Eptesicus* spp. passes in earlier time blocks, and time block order was significant when included as a fixed effect (Table 3).

### Bat call parameters

We extracted echolocation call parameter measurements from 300 *P. pygmaeus* passes (900 individual calls) from five pairs of 5-min time blocks (150 passes per control and treatment blocks). Bandwidth and start frequency were significantly reduced by 5.79 and 5.68 kHz, respectively, during the deterrent treatment (Tables 5, 6), but there was no effect of treatment on call duration, pulse interval (PI), or peak or end frequency (LMMs: duration: χ^2^=0.63, d.f.=1, p=0.43; PI: χ^2^=0.83, d.f.=1, *P*=0.36; peak frequency: χ^2^=0.06, d.f.=1, *P*=0.81; end frequency: χ2=0.30, d.f.=1, *P*=0.58) (see Table S3 for model selection statistics). A higher number of bats in a pass significantly increased both bandwidth (LMM: no. of bats: χ^2^=8.76, d.f.=1, *P*<0.01) and start frequency (LMM: χ^2^=10.58, d.f.=1, *P*<0.01).

**Table 5.**
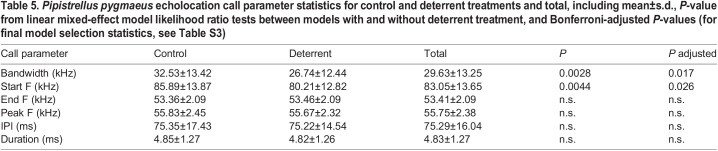
*Pipistrellus pygmaeus* echolocation call parameter statistics for control and deterrent treatments and total, including mean±s.d., *P*-value from linear mixed-effect model likelihood ratio tests between models with and without deterrent treatment, and Bonferroni-adjusted *P*-values (for final model selection statistics, see Table S3)

**Table 6.**
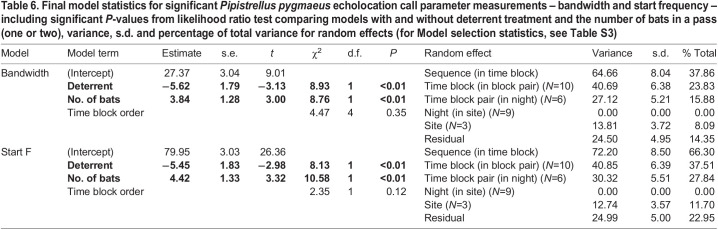
Final model statistics for significant *Pipistrellus pygmaeus* echolocation call parameter measurements – bandwidth and start frequency – including significant *P*-values from likelihood ratio test comparing models with and without deterrent treatment and the number of bats in a pass (one or two), variance, s.d. and percentage of total variance for random effects (for Model selection statistics, see Table S3)

## DISCUSSION

### Do deterrents reduce bat activity, foraging and social behaviour?

As predicted, acoustic deterrents reduced bat activity and feeding behaviour at riparian sites. Overall, bat activity was reduced by 30% during deterrent playback, which is what we would expect for distances up to 30–40 m from the deterrent from previous work at similar sites (Gilmour et al., 2020). Species deterred included *P. pygmaeus*, *Myotis* spp. (likely *M. daubentonii*), and *Nyctalus* spp. and *Eptesicus* spp. (Fig. 3A,C,D). No deterrent effect was found for *P. pipistrellus*; however, previous work at similar sites did find an effect of the same deterrent on this species (Gilmour et al., 2020), so we cannot rule out an effect for this species from this study.

We recorded fewer *P. pygmaeus* feeding buzzes (38%) during deterrent playback, in relation to the general activity of the species (28%), equating to 10% fewer bats still present in the area foraging. However, *P. pygmaeus* continued to social call at an increased rate during deterrent treatment, with 5% more bats still present in the area social calling (23% reduction in calls). Bats deterred from making social calls in the treatment area may still have been in the vicinity and their calls may have still been recorded, reducing the recorded effect of the deterrent on social calls. *Pipistrellus pygmaeus* social calls are louder and of lower frequency than echolocation calls, and so can travel further in the environment and are therefore more likely to be recorded (Middleton et al., 2014). *Pipistrellus pygmaeus* social calls can also have an aversive effect on conspecifics when insect densities are low, in line with the food patch defence hypothesis (Barlow and Jones, 1997). An increase in bats moving into an already occupied patch, outside of the treatment area but close enough to be recorded, could also have decreased the likelihood of detecting a greater effect.

### Do deterrents affect flight performance?

We have shown that as predicted, bats flew significantly faster and with less tortuous flight paths in response to acoustic deterrents (Fig. 2A,B), in line with a reduction in foraging behaviour (Grodzinski et al., 2009). Our results indicate that bats flight paths were similar to those used for commuting, consistent with previous studies that measured bat flight path speed and tortuosity (Holderied et al., 2005; Grodzinski et al., 2009). Bats alter their flight behaviour to optimise aerodynamic costs of flight where possible, but also in line with predation risk, with fast commuting flight more aerodynamically inefficient, but more direct, than slower, more tortuous foraging flight (Grodzinski et al., 2009). The changes in flight behaviour we see during playbacks are therefore in line with reduced foraging, owing to the deterrents. This is supported by the reduction in *P. pygmaeus* feeding buzzes and a shift in the species' echolocation signal structure in response to masking sound of the deterrents recorded.

### Do deterrents affect echolocation call structure?

As predicted, *P. pygmaeus* shifted their echolocation call structure in response to the deterrents (Table 5). The observed reduction in bandwidth and lower start frequency of *P. pygmaeus* echolocation calls in response to deterrents are similar to observed in other studies (Gillam and McCracken, 2007; Bunkley et al., 2015), suggesting the deterrent sound may be acting like other sources of ambient ultrasound, to mask calls, resulting in shift in their signal structure. Owing to the energetic costs of echolocation, bats either focus their energy in a narrower band or spread it over a wider range of frequencies, depending on the situation (Schnitzler and Kalko, 2001). Therefore, by focusing energy into a more narrowband call, bats are more likely to detect echoes against the background noise of the deterrents. Although it would in theory be beneficial to move the echolocation call peak frequency away from the peak of the masker (i.e. the deterrent), shifts in call frequency in the presence of conspecifics only occur in some bat species and not in others (e.g. Ulanovsky et al., 2004). Even where there is a frequency shift, it is not often clear whether this is a jamming avoidance response unless responses to playbacks of conspecific echolocation calls are documented (Gillam et al., 2006). *Pipistrellus pygmaeus* seems to resemble species such as *Taphozous perforatus* (Ulanovsky et al., 2004) in not showing shifts in peak frequency as a jamming avoidance response. A potential confounding factor is that bats will increase their bandwidth when more conspecifics are present in an area, owing to the surrounding airspace representing a more complex environment, similar to clutter (Cvikel et al., 2015; Götze et al., 2016). Therefore, the reduction in start frequency and bandwidth recorded during deterrent periods could be due to fewer bats being present. However, we only considered passes with one or two bats and hence controlled for number of bats in analyses.

Deterrent noise could have also acted as an aversive stimulus as well as a masking one (Luo et al., 2015; Zeale et al., 2016). For example, foraging efficiency of *Myotis daubentonii* was significantly reduced during playbacks of traffic noise that both did and did not overlap with its echolocation call spectral range, but search effort was not affected (Luo et al., 2015). However, as we did not test this hypothesis directly, it is possible that some of the deterrent effect seen in *P. pygmaeus* was due to general aversion. It is also possible that there was some masking effect on calls of other species present at the experimental sites, for example *Myotis* spp., but owing to small sample sizes (<15% of passes), we could not test this.

### Conclusions

In conclusion, we have shown bat activity, foraging behaviour, flight performance and echolocation call structure all change in response to acoustic deterrents. Bats foraged less and flew with more direct flight paths, similar to commuting flight, in the presence of the deterrent sound, likely because of a masking effect on their echolocation calls. The mechanism underpinning acoustic deterrence in bats is therefore likely to be partly due to auditory masking and the impact is a reduction in foraging activity, owing to the masking noise precluding the use of echolocation. Bats foraging in areas such as around wind turbine blades and on or nearby roads are at increased risk of mortality (Mathews et al., 2016; Altringham and Kerth, 2016). Acoustic deterrence could be used to deter bats from these areas and mitigate for the impacts of these structures (Arnett et al., 2013; Gilmour et al., 2020; Weaver et al., 2020). However, as with any mitigation measure, a case-by-case approach is important, weighing up any loss of foraging habitat against the threat of mortality and other potential impact.

## Supplementary Material

Supplementary information
