# Supplementary material for: Acoustic deterrents influence foraging activity, flight and echolocation behaviour of free-flying bats
Source: J Exp Biol. 2021 Oct 28;224(20):jeb242715. doi: 10.1242/jeb.242715 (PMC8601711; doi:10.1242/jeb.242715)
Supplement: Supplementary information [file jexbio-224-242715-s1.pdf]

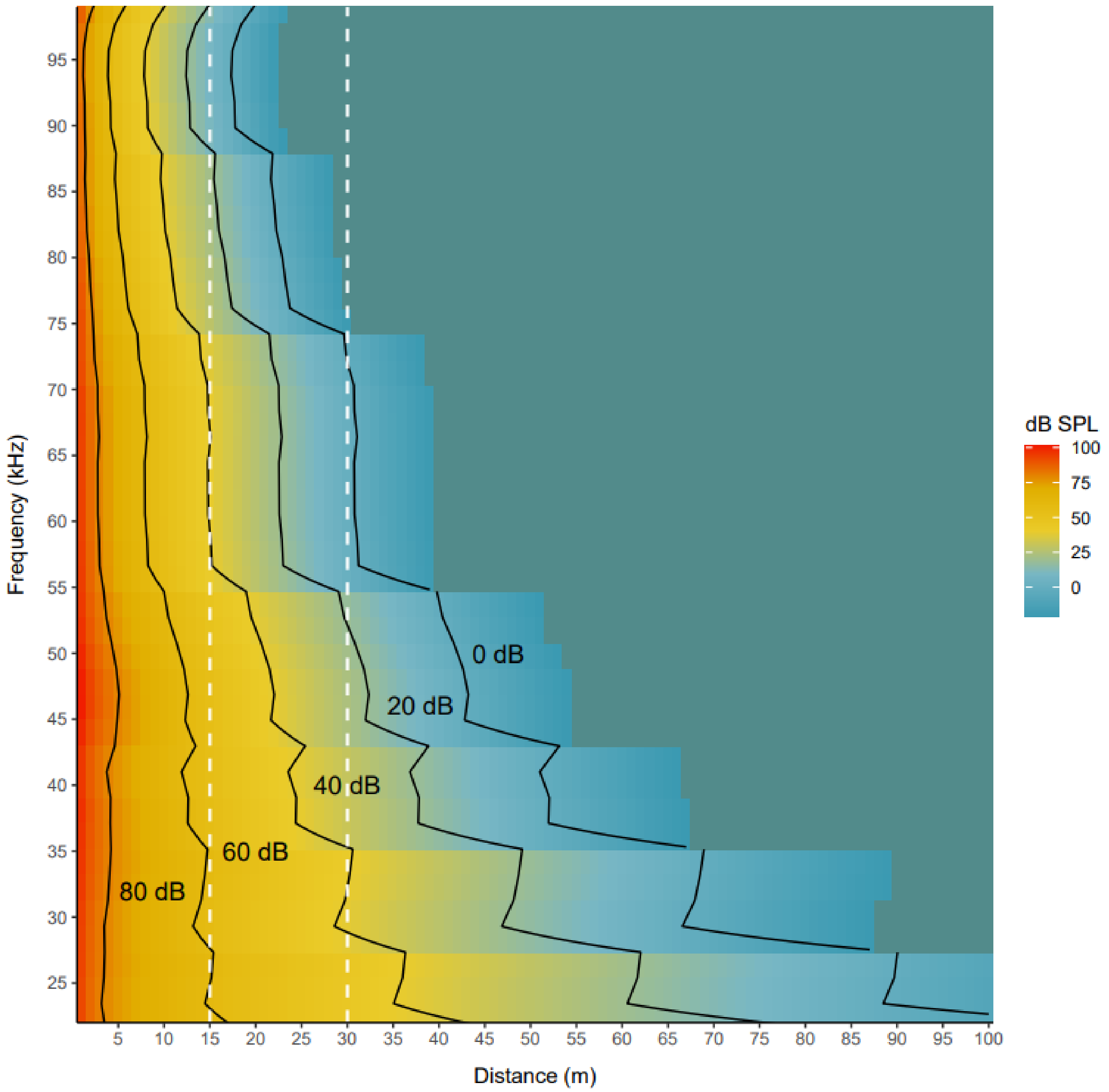

Fig. S1. Heat map of predicted dB SPL (RMS amplitude) for 20-100 kHz broadcast by Deaton deterrent speaker unit over distances of 1-100 m, including contours at 0, 20, 40, 60, 80 dB SPL. White dashed lines indicate fine-scale distance experiment treatment area of 15-30 m. Calculated using calibrated anechoic chamber recordings using spreading loss equations (one way), taking into account atmospheric attenuation and frequency-dependent absorption at 14°C, 90% relative humidity and 101.325 kPa (Bazley 1976; Møhl 1988; Holderied & von Helversen 2003). Teal grey background represents no data (-20 dB SPL and below). Stepped levels due to rounded values for atmospheric attenuation used in calculations.

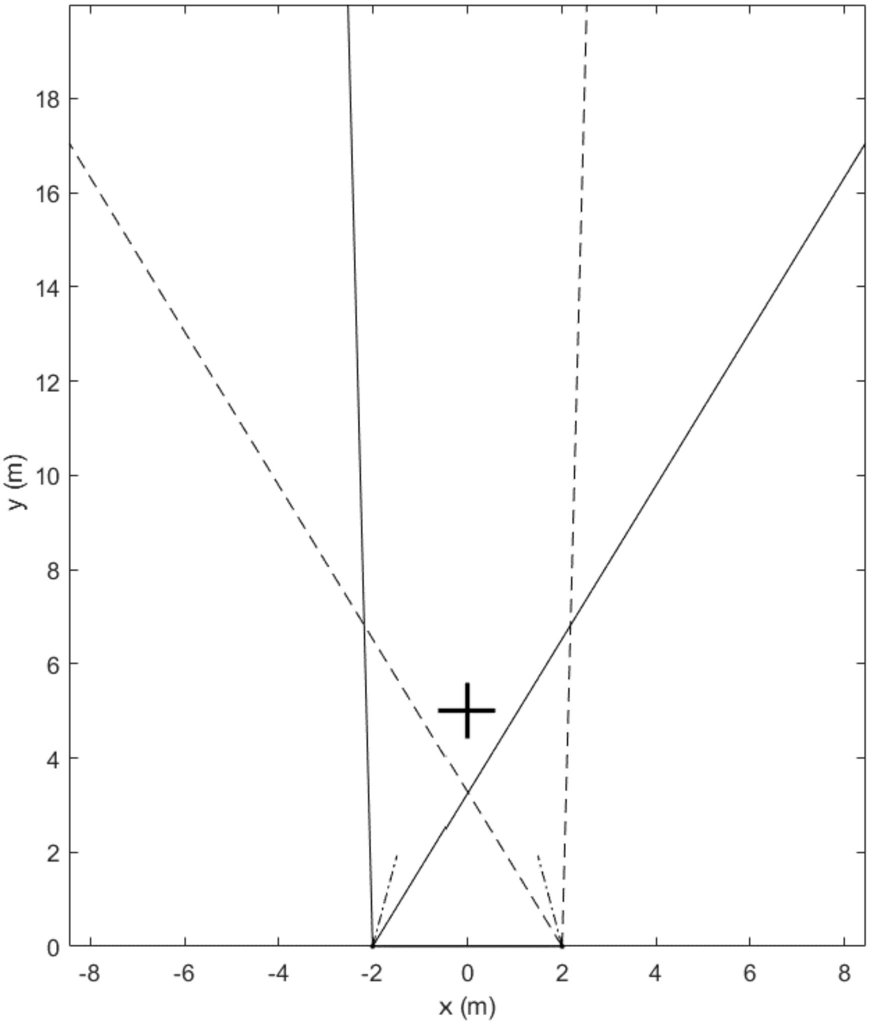

Fig. S2. Calibration set-up, with two thermal imaging cameras with 33° opening angles, positioned at 4 m apart. Camera positioning created a ~4 m wide overlapped field of view (FOV) at 0-20 m. Calibration target represented as a cross plotted at 5 m. Diagram created in MATLAB, to scale.

Journal of Experimental Biology • Supplementary information

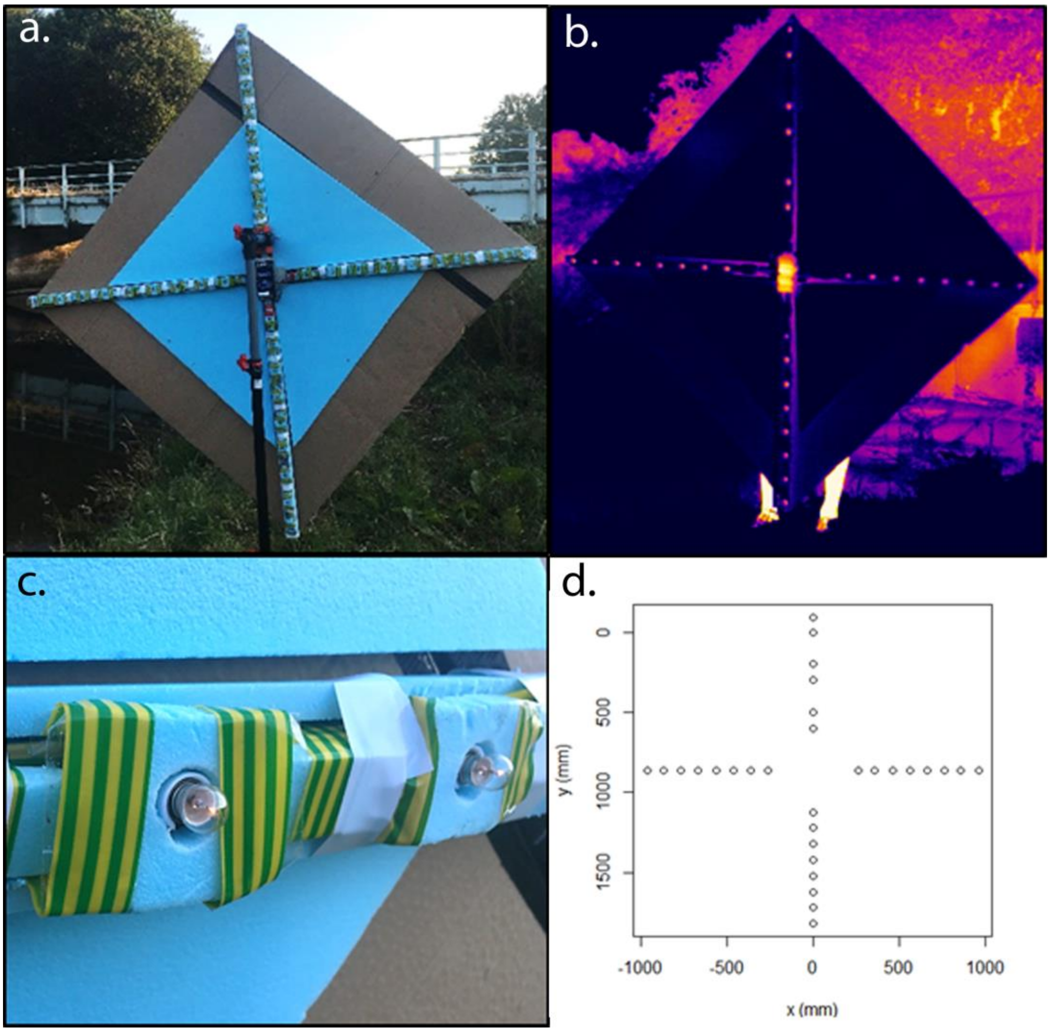

Fig. S3. Images of bespoke calibration target: (a) target in situ at a riparian site, (b) thermal image still of target, (c) close-up of one arm, showing bulbs and insulation and (d) scatter plot showing bulb coordinates used to develop MatchPoint 1.0 software. Photo/image credits L.R.V. Gilmour.

Journal of Experimental Biology • Supplementary information

Table S1. Model selection statistics for trajectory measurements, arranged by AICc weight (AICc Wt) and including number of model parameters (K), cumulative AICc weight (Cum. Wt).

Measurement	Model	Distribution	Link	K	AICc	ΔAICc	AICc Wt	Cum. Wt
**Speed (m/s)**	Deterrent + block number	Gaussian	NA	7	2922.17	0	0.974	0.97
	Deterrent + depth + block number	Gaussian	NA	8	2929.52	7.3	0.025	1
	Deterrent + depth * block number	Gaussian	NA	9	2936.13	14	0.001	1
	Block number	Gaussian	NA	6	2943.11	20.9	0.001	1
	Deterrent	Gaussian	NA	6	2945.36	23.2	0.001	1
	Deterrent + depth	Gaussian	NA	7	2952.64	30.5	0.001	1
	Deterrent * depth * block number	Gaussian	NA	#	2955.43	33.3	0.001	1
**Tortuosity**	Deterrent + distance	cloglog	beta binomial	7	-4371.58	0	0.24	0.24
	Deterrent + distance	logit	beta binomial	7	-4371.31	0.3	0.21	0.45
	Deterrent * distance + block number	cloglog	beta binomial	9	-4371.23	0.4	0.2	0.65
	Deterrent + block number	probit	beta binomial	7	-4370.73	0.9	0.16	0.8
	Deterrent + distance + block number	cloglog	beta binomial	8	-4369.63	1.9	0.09	0.89
	Distance	cloglog	beta binomial	6	-4369.3	2.3	0.08	0.97
	Deterrent	cloglog	beta binomial	6	-4367.61	4	0.03	1
**Distance (m)**	Deterrent	Gaussian	NA	6	6239.36	0	0.868	0.87
	Deterrent + block number	Gaussian	NA	7	6243.69	4.3	0.099	0.97
	Deterrent * block number	Gaussian	NA	8	6246.42	7.1	0.026	0.99
	null	Gaussian	NA	5	6249.01	9.7	0.007	1
**Length (m)**	null	Gaussian	NA	5	9.995264	0	0.971	0.97
	Deterrent	Gaussian	NA	6	17.04132	7	0.029	1
	Deterrent + distance	Gaussian	NA	7	27.00289	17	0	1
	Deterrent + distance + block number	Gaussian	NA	8	38.6426	28.6	0	1
	Deterrent * distance + block number	Gaussian	NA	9	46.47199	36.5	0	1
**Height (m)**	null	Gaussian	NA	5	314.6157	0	0.952	0.95
	Deterrent	Gaussian	NA	6	320.6235	6	0.047	1
	Deterrent + block number	Gaussian	NA	7	329.2744	14.7	<0.001	1
	Deterrent * block number	Gaussian	NA	8	336.5071	21.9	<0.001	1

Journal of Experimental Biology • Supplementary information

Table S2. Model selection statistics for pass data of *Pipistrellus pygmaeus, P.* pipistrellus, *Myotis* spp., *Nyctalus* and *Eptesicus* spp. and P. *pygmaeus* feeding buzz (fb)/social call (sc) data. Models arranged by AICc weight (AICc Wt) and include number of model parameters (K), cumulative AICc weight (Cum. Wt).

Species	Model	link	Distribution	K	AICc	ΔAICc	AICc Wt	Cum. Wt
P. pygmaeus	Deterrent	log	Poisson	6	6723.37	0.00	0.60	0.60
	Deterrent + time block	log	Poisson	5	6724.78	1.40	0.29	0.89
	Deterrent	log	Negative binomial	6	6726.82	3.50	0.11	1.00
	null	log	Poisson	4	6738.70	15.30	0.00	1.00
Myotis spp.	Deterrent + time block	log	Poisson	6	2702.84	0.00	0.73	0.73
	Deterrent + time block	log	Negative binomial	7	2704.84	2.00	0.27	0.99
	Time block	log	Poisson	5	2712.10	9.30	0.01	1.00
	Deterrent	log	Poisson	5	2766.15	63.30	0.00	1.00
P. pipistrellus	Time block	log	Poisson	5	1732.04	0.00	0.58	0.58
	Deterrent + time block	log	Poisson	6	1733.31	1.30	0.31	0.89
	Deterrent + time block	log	Negative binomial	7	1735.33	3.30	0.11	1.00
	Deterrent	log	Poisson	5	1742.20	10.20	0.00	1.00
Nyctalus and *Eptesicus* spp.	Deterrent + time block	log	Poisson	6	572.99	0	0.508	0.51
	Time block	log	Poisson	5	574.735	1.7	0.213	0.72
	Deterrent + time block	log	Negative binomial	7	575.017	2	0.185	0.91
	Deterrent	log	Poisson	5	576.381	3.4	0.094	1.00
P. pygmaeus (fb)	Deterrent + time block	log	Negative binomial	6	3889.00	0.00	0.64	0.64
	Deterrent	log	Negative binomial	7	3890.74	1.70	0.27	0.91
	Deterrent	log	Negative binomial	5	3892.97	4.00	0.09	1.00
	null	log	Poisson	6	3934.89	45.90	0.00	1.00
P. pygmaeus (sc)	Deterrent	log	Negative binomial	6	2540.75	0.00	0.60	0.60
	Deterrent + time block	log	Negative binomial	7	2541.96	1.20	0.33	0.93
	null	log	Negative binomial	5	2545.08	4.30	0.07	1.00
	Deterrent	log	Poisson	6	2782.80	242.10	0.00	1.00

Journal of Experimental Biology • Supplementary information

Table S3. Model selection statistics for bat echolocation call parameter data. Models arranged by AICc weight (AICc Wt) and include number of model parameters (K), cumulative AICc weight (Cum. Wt).

Parameter	Model	Distribution	K	AICc	ΔAICc	AICc Wt	Cum. Wt
Bandwidth	Deterrent + no of bats + time block	Gaussian	10	6202.54	0.00	0.53	0.53
	Deterrent + no of bats	Gaussian	9	6202.80	0.30	0.46	0.99
	Deterrent + time block	Gaussian	9	6211.61	9.10	0.01	1.00
	No of bats	Gaussian	8	6212.70	10.20	0.00	1.00
Peak F	null	Gaussian	7	3366.28	0.00	0.75	0.75
	Deterrent	Gaussian	8	3368.82	2.50	0.21	0.96
	Deterrent + time block + no of bats	Gaussian	10	3373.37	7.10	0.02	0.98
	Deterrent + time block	Gaussian	9	3373.95	7.70	0.02	1.00
Duration	null	Gaussian	7	2139.23	0.00	0.78	0.78
	Deterrent	Gaussian	8	2142.56	3.30	0.15	0.92
	Deterrent + no of bats	Gaussian	9	2143.91	4.70	0.07	1.00
	Deterrent + no of bats + time block	Gaussian	10	2150.72	11.50	0.00	1.00
IPI	Deterrent + time block + no of bats	Gaussian	10	7185.86	0.00	0.55	0.55
	Deterrent + time block	Gaussian	9	7186.98	1.10	0.31	0.86
	null	Gaussian	8	7188.60	2.70	0.14	1.00
	Deterrent	Gaussian	8	7198.11	12.20	0.00	1.00
Start F	Deterrent + no of bats	Gaussian	9	6240.43	0.00	0.50	0.50
	Deterrent + time block + no of bats	Gaussian	10	6240.43	0.00	0.50	0.99
	No of bats	Gaussian	8	6249.57	9.10	0.01	1.00
	Deterrent + block number	Gaussian	9	6251.51	11.10	0.00	1.00
End F	No of bats	Gaussian	8	2892.87	0.00	0.76	0.76
	Deterrent + no of bats	Gaussian	9	2895.27	2.40	0.23	0.98
	Deterrent + time block + no of bats	Gaussian	10	2901.13	8.30	0.01	1.00
	Deterrent + time block	Gaussian	9	2903.84	11.00	0.00	1.00

Journal of Experimental Biology • Supplementary information

References

Bazley EN. 1976. Sound absorption in air at frequencies up to 100 kHz. NPL Acoustics Report Ac 74, National Physical Laboratory, Teddington, England.

Holderied MW, von Helversen O. 2003. Echolocation range and wingbeat period match in aerial-hawking bats. Proceedings of the Royal Society B-Biological Sciences 270:2293-2299.

Møhl B. 1988. Target detection by echolocating bats. In: Nachtigal, PE, & Moore, PWB Animal sonar: processes and performance, Plenum Press, New York, USA 156:435–450.

Journal of Experimental Biology • Supplementary information
